# 

**Published:** 1876-09

**Authors:** 


### Colaborators and Contributors.

GEORGIA.

T. S. Mitchell, M.D.
T. M. Shaw, M.D.
J. N. Sheny, M.D.
J. M. Tules, M.D.
G. D. Case, M.D.
W. H. Hall, M.D.
S. G. White, M.D.
J. C. Wisdom, M.D.
J. E. Pope, M.D.
E. H. W. Hunter M.D.
Thomas Burdell, M.D.
L. B. Bouchelle, M.D.
W. C. Musgrove, M.D.
W. L. M. Harriss, M.D.
J. M. Tullis, M.D.
G. S. Ullman, M.D.
John Coster, M.D.

KENTUCKY.

Charles Kearns, M.D.
W. S. Taylor, M.D.
Douglass Morton, M.D.

TEXAS.

L. R. Lincecum, M.D.
T. J. Heard, M.D.

TENNESSEE.

F. K. Bailey, M.D.
F. A. Ramsey, M.D.
J. D. Tucker, M.D.

WEST VIRGINIA.

I. . Berry, M.D.
N. D. Tobey, M.D.
P. H. Clark, M.D.
A. L. Knight, M.D.

IOWA.

C. H. Tidd, M.D.
Charles H. Lathrop, M.D.

ALABAMA.

J. J. Grace, M.D.
J. D. Rush, M.D.
A. C. Edwards, MD.
S. B. Hogan, M.D.
J. B. Barnett, M.D.
J. C. Knox, M.D.

NORTH CAROLINA.

R. S. Hallsey, M.D.
John W. Booth, M.D.
E. A. Anderson, M.D.
George A. Foot M.D.
H. Kelly, M.D
A. B. Pierce, M.D.
S. S. Satchwell, M.D.
J. B. Hughs, M.D.
Patrick Booth, M.D.

SOUTH CAROLINA.

G. M. Rivers, M.D.
E. T. McSwain, M.D.

OHIO.

W. P. Wells, M.D.
J. C. Bishop, M.D.
E. H. Trickle, M.D.
W. W. Fierce, M.D.
J. B. Graves, M.D.
J. M. Lucas, M.D.

NEW YORK.

J. S. Baily, M.D.
Ely Van DeWalker, M.D.
Lewis Balch, M.D.
W. H. Vail, M.D.

OREGON.

W. W. Cusick, M.D.

NEW JERSEY.

P. A. Harriss, M.D.
Jno. D. McGill, M.D.
D. M. Forman, M.D.

VIRGINIA.

J. N. Upshur, M.D.
L. B. Anderson, M.D.
W. F. Barr, M.D.
E. A. Drewry, M.D.
S. P. Ripley, M.D.
J. S. Wharton, M.D.
R. J. Preston, M.D.
E. Horner, Jr., M.D.

MISSISSIPPI.

E. Cutter, M.D.
T. H. Mayo, MD.
I. H. Payne, M.D.
J. M. Lewis, M.D.
A. G. Smythe, M.D.
W. T. Beall, M.D.
W. L. Lipscomb, M.D.
Robert Kells, M.D.
E. T. Henry, M.D.
T. P. Lockwood, M.D.
S. V. D. Hill, M.D.
Edward Lee, M.D.

ARKANSAS.

C. D. Hodge, M.D.
A. W. Hobson, M.D.
J. K. Hodge, M.D.
A. D. Hodge, M.D.

INDIANA.

A. Patton, M.D.
G. A. Dyer, M.D.
J. M. Williamson, M.D.

ILLINOIS.

John W. Weir, M.D.

MARYLAND.

A. F. Erich, M.D.

LOUISIANA.

J. W. Wimbish, M.D.

MICHIGAN.

A. E. Hoadley, M.D.

